# Oral Local Anesthesia Successfully Ameliorated Neuropathic Pain in an Upper Limb Suggesting Pain Alleviation through Neural Plasticity within the Central Nervous System: A Case Report

**DOI:** 10.1155/2011/984281

**Published:** 2011-05-22

**Authors:** Jun Hozumi, Masahiko Sumitani, Arito Yozu, Toshiya Tomioka, Hiroshi Sekiyama, Satoru Miyauchi, Yoshitsugu Yamada

**Affiliations:** ^1^Department of Anesthesiology and Pain Relief Center, The University of Tokyo Hospital, 7-3-1 Hongo, Bunkyo-ku, Tokyo 113-0033, Japan; ^2^Department of Rehabilitation Medicine, Graduate School of Medicine, The University of Tokyo, Tokyo, Japan; ^3^Kobe Advanced ICT Research Center, National Institute of Information and Communications Technology, Kobe 651-2492, Japan

## Abstract

Neural blockades are considered an alternative to pharmacotherapy for neuropathic pain although these blockades elicit limited effects. We encountered a patient with postbrachial plexus avulsion injury pain, which was refractory to conventional treatments but disappeared temporarily with the administration of the local anesthetic lidocaine around the left mandibular molar tooth during dental treatments. This analgesic effect on neuropathic pain by oral local anesthesia was reproducible. Under conditions of neuropathic pain, cerebral somatotopic reorganization in the sensorimotor cortices of the brain has been observed. Either expansion or shrinkage of the somatotopic representation of a deafferentated body part correlates with the degree of neuropathic pain. In our case, administration of an oral local anesthetic shrank the somatotopic representation of the mouth, which is next to the upper limb representation and thereby expanded the upper limb representation in a normal manner. Consequently, oral local anesthesia improved the pain in the upper limb. This case suggests that pain alleviation through neural plasticity within the brain is related to neural blockade.

## 1. Introduction

Neuropathic pain typically appears following peripheral nerve injury due to neuropathies, plexopathies, and trauma to selected sites within the central nervous system (CNS). Recently, evidence-based recommendations of pharmacological treatments for neuropathic pain have been proposed based on both positive and negative results from multiple randomized controlled trials. However, approximately 10–15% of all neuropathic pain patients are refractory to pharmacotherapy. For these cases, more invasive pain-management interventions, such as intrathecal drug delivery, neurostimulation, or neural blockade, may be used. Ideally, blocking neural transmission, either temporarily by using local anesthetics or permanently by surgical nerve ablation, can reduce neuropathic pain; however, no neural blockades have been found to be consistently successful [1]. Here, we report on a case of a patient with postbrachial plexus avulsion injury pain whose neuropathic pain had been refractory to several evidence-based pharmacotherapies and interventions, such as spinal cord stimulation, cervical epidural blockade, and brachial plexus blockade. His pain could be well controlled by oral local anesthesia, suggesting pain alleviation through neural plasticity within the CNS.

## 2. Case Report

A 49-year-old man, who had a left brachial plexus avulsion injury 10 years before, experienced severe neuropathic pain in his left upper limb immediately after the trauma. The patient complained of continuous burning, pressing, and tingling pain in the upper limb. From the beginning of the perception of the pain in his upper limb, he felt illusory perceptions of fingers touching his face although he did not perceive pain or any other sensory deficits in the face. He had been treated several times for the pain through left brachial plexus blockades and cervical epidural blockades, with no success. His neuropathic pain decreased slightly when taking pregabalin and with the application of cervical spinal cord stimulation (SCS), but it remained severe. He did not have any pain or trigger areas in the face getting caries of the teeth. He once underwent a dental treatment for his left mandibular molar tooth. When local anesthesia was applied around the left mandibular molar tooth (3 mL, 0.5% lidocaine), he felt the enlargement of that region, which was followed by an immediate disappearance of his neuropathic pain. At that time, the illusory finger sensations in the face disappeared. Approximately 2 hours after the dental treatment, the neuropathic pain returned and gradually increased to predental treatment levels. A nonsteroidal anti-inflammatory drug, loxoprofen, completely ameliorated the dental pain but was not effective against the neuropathic pain. Since then, the patient had 3 dental treatments, and local anesthesia around the left molar tooth consistently ameliorated his neuropathic pain. Analgesic effects consistently lasted for several hours following the administration of the local anesthesia. His neuropathic pain was able to be mildly controlled by a combination of pregabalin, SCS, and local anesthesia around the left molar tooth although the molar tooth had completely improved. The use of oral local anesthesia for breakthrough neuropathic pain had been especially effective.

We obtained the patient's consent to report his progress, in accordance with the Declaration of Helsinki.

## 3. Discussion

**Figure 1:**
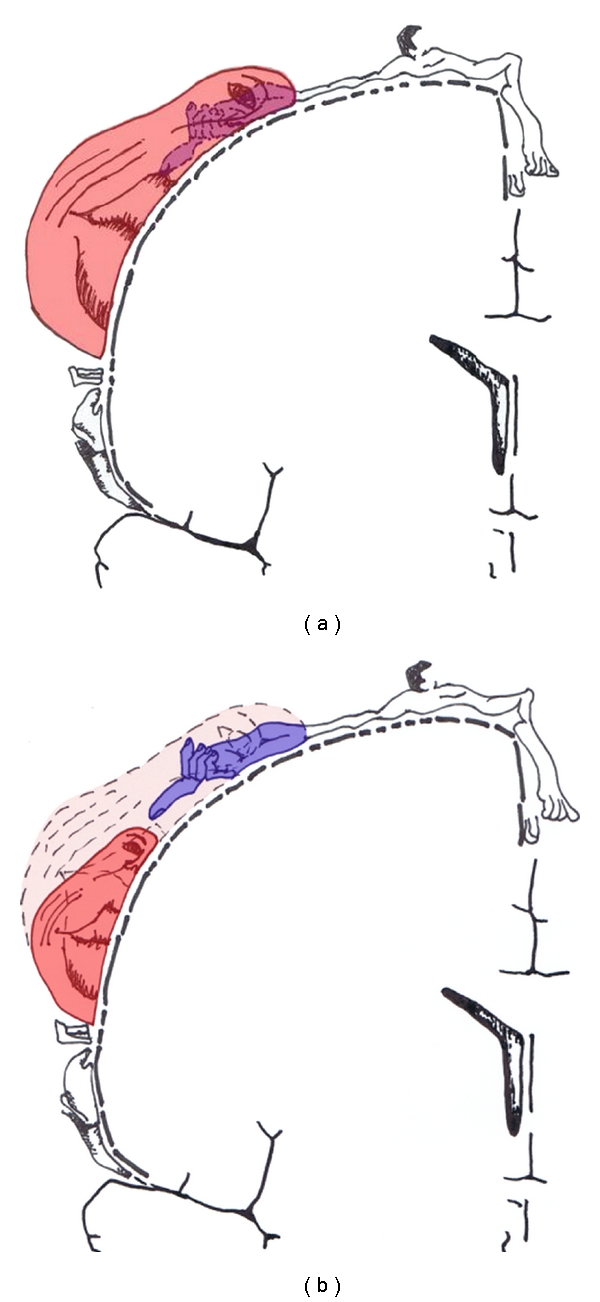
Topographical somatotopic reorganization in the sensorimotor cortices following deafferentation by a brachial plexus avulsion injury (a) and normalization of the reorganization by application of local anesthesia in the mouth (b).

Under conditions of neuropathic pain, particularly for deafferentation pain following massive nerve injury, such as postamputation phantom limb pain, postbrachial plexus injury pain, or postspinal cord injury pain, cerebral somatotopic reorganization in the sensorimotor cortices of the brain is observed. Following deafferentation of an upper limb by nerve injury, the somatotopic region corresponding to the upper limb in the sensorimotor cortices shrinks, and the somatotopic region responding to the facial region, which is located next to the upper limb, expands (Figure 1(a)) [2, 3]. The degree of shrinkage of the upper limb representation correlated linearly with the severity of the neuropathic pain [4]. Further, expansion of the somatotopic representation of the affected body part correlated with pain alleviation through neurorehabilitation techniques [5–7]. Therefore, somatotopic reorganization in the sensorimotor cortices closely relates to pathophysiological mechanisms underlying neuropathic pain and its alleviation.

Concerning the somatotopic reorganization of the face and hand regions, the overlapping of these regions can sometimes induce the following illusion in patients with a deafferentation of a hand: touching the face creates obvious referred sensation of fingers in the face as if the fingers are embedded in the face [8]. We consider one possibility that the analgesic effects of the oral local anesthesia in our case were derived from the neural plasticity in the sensorimotor cortices because our patient perceived a similar illusory sensation of fingers in the face. Deafferentation by local anesthesia, as well as that by nerve injury, shrinks the somatotopic representation of the exposed body part and simultaneously expands the nearby somatotopic representation in the sensorimotor cortices, and these are not associated with subcortical changes [9, 10]. On the basis of this notion, we speculated that, in our case, local anesthesia in the mouth shrank the mouth/face representation and subsequently expanded the somatotopic representation of the hand/upper limb within the sensorimotor cortices (Figure 1(b)), resulting in amelioration of the neuropathic pain in the upper limb. The disappearance of the illusory finger sensations in the face soon after the oral local anesthesia supported the intimate relationship between analgesic effects of the upper limb pain and cerebral reorganization of hand/upper limb and face/mouth representations.

In general, neural blockades are applied to painful body parts in order to block neural transmission; however, the clinical significance of neural transmission blockades remains unclear for nerve-injured neuropathic pain because of deafferentation. Local anesthesia at an intact limb contralateral to the painful limb has been reported to display clear analgesic effects on postamputation phantom limb pain, suggesting pain alleviation through neural plasticity within the CNS [11]. Thus, several types of local anesthesia or neural blockades on unaffected body parts have distinct clinical significance compared to neural transmission blockades, whereas peripheral nerve blockades shrink the somatotopic area of the exposed body part and seem to have no analgesic effect on neuropathic pain in general. Specific analgesic effects on neuropathic pain from local anesthesia and neural blockade could be derived from CNS plasticity. In the future, functional brain imaging studies examining the relationship between neural blockade application for neuropathic pain and CNS plasticity need to be performed in order to better understand somatotopic reorganization in the sensorimotor cortices induced by neural blockades.

## 4. Conclusion

For neural blockades, oral local anesthesia is a novel candidate for treating neuropathic pain in the upper limb, and the analgesic effect might be derived from its effects on neural plasticity within the CNS.
